# Renal function changes in patients with subclinical hyperthyroidism: a novel postulated mechanism

**DOI:** 10.1007/s12020-023-03361-3

**Published:** 2023-07-25

**Authors:** Magdy Mohamed Allam, Hanaa Tarek El-Zawawy, Tarek Hussein El-Zawawy

**Affiliations:** 1 Internal Medicine Department, Endocrinology division, Alexandria University Student Hospital, Alexandria, Egypt; 2 Internal Medicine Department, Endocrinology division, Faculty of Medicine, Alexandria University, Alexandria, Egypt; 3 Cardiology and Angiology Department, Faculty of Medicine, Alexandria University, Alexandria, Egypt

**Keywords:** Hyperthyroidism, Renal function, Hypertension, Renal Arterial distensibility

## Abstract

**Background:**

Subclinical hyperthyroidism (SCH) is found to be associated with renal dysfunction. Hyperthyroidism is a well-known cause of secondary systolic hypertension. However, the effect of SCH on the kidney and its vasculature is still unknown.

**Aim:**

To assess the presence of renal function changes and renal vasodysfunction in SCH patients and their relation to hypertension.

**Methods:**

The study included 321 patients with SCH and 80 healthy matched controls. Laboratory investigations included thyroid function tests, anti-TSH receptor antibody (TRAb), creatinine, estimated glomerular filtration rate (eGFR), serum osmolarity (S. Osmol), urine osmolarity (U. Osmol), Fractional Excretion of Sodium (FeNa), Fractional Excretion of Potassium (FeK), copeptin (CPP), and aldosterone/renin ratio (ARR). Ultrasound for the thyroid gland, echocardiography, total peripheral resistance (TPR), flow-mediated dilatation (FMD), and Renal Arterial distensibility (RAD) was also done.

**Results:**

Serum creatinine was significantly lower while eGFR was significantly higher in SCH patients compared to euthyroid subjects (mean 0.59 ± 0.11 mg/dl Vs mean 0.8 ± 0.1 mg/dl, *p* = 0.001 and mean 128.28 ± 14.69 ml/min/1.73m2 Vs mean 100.49 ± 14.9 ml/min/1.73m2, *p* = 0.013, respectively). The TPR and FMD showed a significant decrease in SCH group compared to controls (mean 975.85 ± 159.33 mmHg.min/L Vs mean 1120.24 ± 135.15 mmHg.min/L, *p* = 0.045 and mean 7.03 ± 4.02% Vs mean 13.48 ± 4.57%, *p* = 0.003, respectively). RAD was significantly higher in hypertensive SCH patients compared to normotensive SCH patients (mean 17.82 ± 2.46 mmHg Vs mean 11.98 ± 3.21 mmHg, *p* = 0.001).

**Conclusion:**

SCH patients showed vascular resistance reduction. Alterations in thyroid hormones and blood pressure could be the driving mechanisms for the change in renal functions in patients with SCH.

## Introduction

Thyroid disorders are very common with a high prevalence among the general population. Thyroid diseases are associated with many detrimental effects that have a serious impact on various body systems [1].

Hyperthyroidism is a form of thyroid disease in which excess thyroid hormones are synthesized and secreted by the thyroid gland. This is distinguished from thyrotoxicosis which refers to the clinical hypermetabolic syndrome resulting from serum elevation in thyroid hormone levels that could be either due to hyperthyroidism or extra thyroidal sources of thyroid hormone secretion. High thyroid radioactive iodine uptake is pathognomonic to hyperthyroidism [2].

Hyperthyroidism can be overt or subclinical. Overt hyperthyroidism is defined as low serum thyroid-stimulating hormone (TSH) and elevated serum thyroxine (T_4_), tri-iodothyronine (T_3_), or both. Whereas subclinical hyperthyroidism (SCH) is defined as low serum TSH level with normal serum levels of T3 and T4. The prevalence of SCH ranges from 0.6 to 1.8% in adults [3]. According to the Third National Health and Nutrition Examination Survey (NHANES III), 0.7% out of 16,533 people were reported to have SCH with TSH < 0.1 mU/L) [4]. Although SCH is an early stage of hyperthyroidism, nevertheless patients with SCH are at increased risk of cardiovascular disease and endothelial dysfunction [5].

The relationship between thyroid hormones and kidney function is well-known for many years. Thyroid diseases adversely affect renal physiology, meanwhile, kidney diseases could result in thyroid dysfunction. Thyroid hormones contribute to the maintenance of water and electrolyte balance, and participate in the renal transport system. Hyperthyroidism results in an increased glomerular filtration rate (GFR) by about 18–25% due to increased renal blood flow and activation of renin-angiotensin-aldosterone system (RAAS). Also, these changes have been reported in patients with subclinical hyperthyroidism [6–8] Increased RAAS activity results in afferent arteriolar vasodilatation and efferent arteriolar vasoconstriction with a consequent increase in the filtration pressure and hypoperfusion of the proximal convoluted tubules (PCT) with avid sodium and chloride reabsorption. In addition, hyperthyroidism results in increased activity of the basolateral Na/K ATPase, apical Na – H exchanger (NHE), and the Na – Pi co-transporter which increases the proximal sodium reabsorption [9]. This ultimately results in renal vasodysfunction, defined as impaired renal vasodilation and abnormally increased renal vascular resistance in response to increased salt retention which is involved in the initiation of salt-induced hypertension [10]. Regarding the effect of subclinical hyperthyroidism (SCH) on kidney function, data are still limited and controversial [7, 11, 12].

Hyperthyroidism is a well-known cause of secondary systolic hypertension. Hyperthyroidism can cause hypertension through increased cardiac output as well as increased levels of renin, angiotensin, and aldosterone [13]. Yet, the relationship between SCH and hypertension is still a matter of ongoing debate. Multiple studies have investigated the association of SCH with hypertension; some studies demonstrated that subjects with SCH had an increased risk of hypertension [14] while others did not find such an association [15, 16]. Thus, it was interesting to study renal changes in patients with SCH that may be a probable cause of hypertension in those patients.

## Aim of the work

This study aims to assess the presence of renal function changes and renal vasodysfunction in patients with subclinical hyperthyroidism and their relationship to hypertension in those patients.

## Patients and methods

### Patients

To achieve 90% power with target significance level at 5% using one sample *t*-test power analysis in this cross-sectional study, we enrolled 321 patients with SCH (under the age of 60 years) and 80 healthy age and sex-matched adults. Patients were recruited from the Endocrinology outpatient clinics at Alexandria Main University Hospital and Alexandria University Students’ Hospital between January 2017 and January 2020.

Exclusion criteria were pregnancy, chronic liver failure, chronic heart failure, ischemic heart disease, past history of hypertension, cerebrovascular disease, peripheral vascular disease, atrial fibrillation, receiving medications known to affect renal function or vasculature such as (contrast agents, nonsteroid anti-inflammatory drugs, antibiotics, ACE related drugs and diuretics), urinary tract infection, diabetes insipidus, diabetes mellitus, and adrenal insufficiency.

### Methods

All patients and controls were subjected to:

History taking with emphasis on smoking status and the use of drugs.

Clinical examination including measurement of body mass index (BMI), which was calculated as BMI = Body weight (Kg)/Height (m^2^).

The thyroid function was evaluated by Free T3 (FT3) (normal 2.2–5.2 pg/ml), Free T4 (FT4) (normal 0.85–1.9 ng/dl), and TSH (normal 0.4–4.2 mIU/l) using electrochemiluminescence immunoassay (Cobas 6000, Roche Diagnostics). Subclinical hyperthyroidism was defined as a low TSH level below 0.4 mIU/l with normal free T3 and free T4 levels [17]. Graves’ disease was diagnosed based on positive TSH Receptor Antibody (TRAb) (TRAb ≥ 1.5 IU/L). Also, radioactive iodine uptake was conducted in some case to differentiate other types of thyroiditis and toxic nodular goiter.

Ultrasound for the thyroid gland was done to assess thyroid morphology using Kontron device with liner probe, 7.5–10 MHz transducer.

Other laboratory tests included blood urea nitrogen (BUN), creatinine, estimated glomerular filtration rate (eGFR), serum osmolarity (S. Osmol), urine osmolarity (U. Osmol), Fractional Excretion of Sodium (FeNa), Fractional Excretion of Potassium (FeK), Copeptin (CPP), and Aldosterone/Renin ratio (ARR).

- eGFR (ml/min/1.73 m^2^) was calculated using the Chronic Kidney Disease Epidemiology Collaboration equation (CKD-EPI) [18]
  eGFR = 141 × min (Scr/k)^a^ × max (Scr/k)^−1.209^ × 0.993^age^ × 1.018 [if female] × 1.159 [if black]
- FENa (%) [19] = 100 × (serum creatinine × urine sodium)/(serum sodium × urine creatinine)
- FeK (%) [19] = 100 × (serum creatinine × urine Potassium)/(serum Potassium × urine creatinine)
- Serum osmolarity = (2 × (Na+K)) + (glucose/18) + (BUN/2.8)
- Urine osmolarity was detected by osmometer.

**Blood pressure (BP)** was assessed twice with 2 weeks apart. Systolic hypertension (HTN) was diagnosed according to the criteria of the JNC 8: defined as systolic blood pressure (SBP) ≥ 140 mmHg and diastolic blood pressure (DBP) < 90 mmHg.

**Echocardiography** [20, 21]: Using HD11XE echo machine (Philips) to assess:

Cardiac output (CO) = Heart rate (HR) X Stroke volume (SV) [SV = EDV-ESV], Ejection fraction (EF), Fractional shortening (FS), Early diastolic velocity (E),Late diastolic velocity (A), and E/A

**Total peripheral resistance (TPR)** (mmHg.min/L) [22] = Mean arterial BP/CO

**Flow-mediated dilatation (FMD)** (%) of the brachial artery was carried out using B-mode ultrasound to assess the endothelial function: a sphygmomanometer blood pressure cuff was placed above the antecubital fossa and baseline rest ultrasonographic image of the brachial artery diameter was acquired. Thereafter, arterial occlusion was created by means of cuff inflation to 50 mmHg supra systolic pressure to occlude arterial inflow for 5 min. Another image was obtained 60 s after cuff release. Reactive hyperaemia that occurs after cuff release was determined by the mosaic change in color-flow imaging and increase in flow volume. FMD was calculated as the percentage change in brachial artery diameter in response to hyperaemia. Results of FMD in patients with SCH were assessed in relation to controls taking into consideration that the cutoff value for normal endothelial function assessed by FMD of the brachial artery is 7.1% [23].

**Renal Arterial distensibility (RAD)**, the local measurement of arterial stiffness, is expressed in terms of the relative change in the arterial volume for given pressure changes, and this is the inverse of the elastic modulus. RAD was obtained using the distensibility coefficient (DC) in mmHg-1 [DC = {Dmax^2^-Dmin^2^}/D^2^ X PP] (Dmax and Dmin are the maximum and minimum arterial diameters for the pressure changes, respectively and PP is pulse pressure). Participants were kept sitting in a quiet room for 15 min before measuring arterial stiffness. RAD was determined by acquiring waveforms at the right and left renal arteries. Three measurements were acquired from each kidney, and the mean values for right and left kidneys were recorded for further analysis [24].

### Statistics

Statistical analyses were performed using SPSS for Windows (SPSSInc., Chicago, IL, USA), version 25. The continuous variables were expressed as the mean ± standard deviation and compared using an unpaired *t*-test & Mann Whitney test. Categorical variables were expressed as a number (percentage) and compared using a *χ*2 test.

Multivariable models for several factors affecting GFR were adjusted for age, sex, BMI and smoking index to avoid possible confounding factors. The multivariable models were additionally adjusted for SBP, diastolic blood pressure, mean arterial pressure (MAP), smoking status, fasting blood glucose and BMI. We performed an analysis of covariance where mean values of decline in eGFR, FeNa, and FeK were compared across categories of endothelial function and blood pressure.

Analysis of covariance (ANCOVA) was performed to compare the significant parameters (*p* < 0.05) in univariate analysis between the patients with SCH and euthyroid controls after adjustments for the potential confounding factors. The adjusted mean (±standard error) was provided by the estimated marginal means in the ANCOVA analysis. A statistically significant interaction, we reported the simple main effects between blood pressure and endothelial dysfunction (at *p* ≤ 0.025). The linear relationship between each variable and the levels of GFR were examined. Univariate and multivariate linear regression analyses were used. The multivariate linear regression model for GFR was applied to find the independent determinants among the candidate variables (*p* < 0.10 in the univariate analysis) after adjustments for the potential confounding factors and quantified by the standardized regression coefficient (β).

## Results

### Demographic data and thyroid function & morphology results of patients with SCH and euthyroid controls

Out of 321 patients with endogenous SCH; 261 patients had Graves’ disease (based on TRAb positivity) and 60 patients had toxic multinodular goitre (based on TRAb negativity, nodularity of the gland in the ultrasound, and high radioiodine scan uptake). Patients and euthyroid controls were well matched. The mean age in the SCH group was 43 ± 11.46 years while in the euthyroid group was 39.63 ± 12.83 years. Most of the studied subjects were females; accounting for 57.14% in the SCH group and 62.5% in the euthyroid group. In the SCH group, the mean BMI was 22.5 ± 2.1 Kg/m^2^ and 16.3% of the patients were smokers, whereas in the euthyroid group; the mean BMI was 24.6 ± 3.7 Kg/m^2^ and 13.8% of the subjects were smokers [Table 1].

**Table 1:** Demographic data and thyroid function & morphology of the studied groups

	SCH group (*n* = 321)	Euthyroid group (*n* = 80)	*P*
Patients’ characteristics			
Age (years)	43 ± 11.46	39.63 ± 12.83	0.462
Sex (female %)	57.14	62.5	0.813
BMI (Kg/m^2^)	22.5 ± 2.1	24.6 ± 3.7	0.840
Smokers (%)	16.3	13.8	0.20
Thyroid function			
Free T3 (pg/ml)	4.94 ± 0.18	2.83 ± 0.63	^a^0.0001
Free T4 (ng/dl)	1.76 ± 0.1	1.23 ± 0.23	^a^0.0001
TSH (μIU/ml)	0.01 ± 0.01	1.87 ± 0.98	^a^0.0001
Thyroid morphology			
Gland volume (ml)	22.3 ± 10.04	4.76 ± 1.79	^a^0.0001
Vascularity (%)			
Normal	14.29	100	^a^0.0001
G1 Hypervascularity	14.29	0	
G2 Hypervascularity	71.42	0	
Echogenicity (%)			
Isoechoic	42.85	56.25	^a^0.026
Hyperechoic	14.29	43.75	
Hypoechoic	42.86	0	

*P* *p* value for comparing between the studied groups

*n* Number of patients

*SCH* Subclinically hyperthyroid

*BMI* Body mass index

^a^Statistically significant at *p* ≤ 0.05

### Renal function results

Table (2) shows that mean serum creatinine was significantly lower in SCH patients compared to euthyroid subjects (mean 0.59 ± 0.11 mg/dl Vs mean 0.8 ± 0.1 mg/dl, *p* = 0.001). Mean eGFR was significantly higher in SCH patients than in euthyroid controls (mean 128.28 ± 14.69 ml/min/1.73 m^2^ Vs mean 100.49 ± 14.9 ml/min/1.73m^2^, *p* = 0.013). Mean BUN was significantly higher in SCH group than in the control group (mean 16.29 ± 3.4 mg/dl Vs mean 13.06 ± 2.08 mg/dl, *p* = 0.012). Yet, there was no significant difference between SCH group and euthyroid controls regarding; S. Osmol, U. Osmol, serum Na + , Urine Na + , FENa %, serum K + , urine K + , FEK %, Renin/Aldosterone ratio, S/U(Na/K), and CPP.

**Table 2:** Results of the renal function tests, blood pressure, and indices of arterial stiffness in the studied groups

	SCH (*n* = 321)	EUTHYROID (*n* = 80)	*P*
Creatinine (mg/dl)	0.59 ± 0.11	0.8 ± 0.1	^a^0.001
eGFR (mL/min/1.73m2)	128.28 ± 14.69	100.49 ± 14.9	^a^0.013
BUN (mg/dl)	16.29 ± 3.4	13.06 ± 2.08	^a^0.012
S. Osmol (mOsmol/L)	290.23 ± 3.15	288.8 ± 6.22	0.640
U. Osmol (mOsmol/L)	571.43 ± 179.95	600 ± 193.22	0.778
Na + (mmol/L)	140.14 ± 1.46	139.88 ± 3.12	0.893
Urine Na + (mEq/L)	99.43 ± 46.52	75.14 ± 31.82	0.229
FENa (%)	2.47 ± 2.36	2.27 ± 1.07	0.789
K + (mmol/L)	4.36 ± 0.38	4.24 ± 0.34	0.417
Urine K + (mEq/L)	41.85 ± 21.77	48.59 ± 20.48	0.285
FEK (%)	33.75 ± 18.6	48.7 ± 24.6	0.109
Renin/Aldosterone ratio	14.36 ± 3.05	14.96 ± 3.08	0.640
CPP (pmol/L)	2.02 ± 1.83	3.09 ± 2.23	0.256
Hypertensive (%)	28.97 (n = 93)	0	^a^0.029
SBP (mmHg)	130 ± 13.85	117.81 ± 5.47	^a^0.002
DBP (mmHg)	72.86 ± 8.09	73.13 ± 4.79	0.939
Mean BP (mmHg)	91.91 ± 7.54	88.03 ± 3.71	0.139
Pulse (BPM)	94.86 ± 15.25	78.63 ± 5.37	^a^0.003
CO (l/min)	7.79 ± 2.88	6.37 ± 0.84	^a^0.027
TPR (mmHg.min/L)	975.85 ± 159.33	1120.24 ± 135.15	^a^0.045
EF (%)	72.86 ± 3.67	68.19 ± 4.51	^a^0.022
FS (%)	40.48 ± 2.85	33.9 ± 5.86	^a^0.008
E/A	1.41 ± 0.25	1.3 ± 0.29	0.229
Subjects with abnormal FMD (%)	85.71	1	^a^0.000
FMD (%)	7.03 ± 4.02	13.48 ± 4.57	^a^0.003
RAD (mmHg^−1^)	9.83 ± 2.83	12.71 ± 4.29	^a^0.010

*P* *p* value for comparing between the studied groups

*n* Number of patients, *SCH* Subclinically hyperthyroid, *eGFR* Estimated glomerular filtration rate, *BUN* Blood urea nitrogen, *S. osmol* Serum osmolarity, *U. osmol* Urine osmolarity, *FENa* Fractional extraction of sodium, *FEK* Fractional extraction of potassium, *CPP* Copeptin, *SBP* Systolic blood pressure, *DBP* Diastolic blood pressure, *CO* Cardiac output, *TPR* Total peripheral resistance, *EF* Ejection fraction, *FS* Fractional shortening, *E/A* Early diastolic velocity/Late diastolic velocity, *FMD* Flow mediated dilatation, *RAD* Renal artery distensibility

^a^Statistically significant at *p* ≤ 0.05

### Comparing BP and indices of arterial stiffness in SCH and control groups

Ninety-three patients (28.97%) with SCH were hypertensive, while none of the euthyroid group was hypertensive. The mean SBP was significantly higher in SCH group compared to control group (mean 130 ± 13.85 mmHg Vs mean 117.81 ± 5.47 mmHg, *p* = 0.002), while there was no significant difference in DBP and mean BP. The mean pulse was significantly higher in SCH patients compared to control group (mean 94.86 ± 15.25 BPM Vs mean 88.03 ± 3.71 BPM, *p* = 0.003). The mean CO was significantly higher in SCH group compared to control group (mean 7.79 ± 2.88 L/min Vs mean 6.37 ± 0.84 L/min, *p* = 0.027). The mean EF and FS were significantly higher in SCH group in comparison to control group (mean 72.86 ± 3.67% VS mean 68.19 ± 4.51%, *p* = 0.022 and mean 40.48 ± 2.85% Vs mean 33.9 ± 5.86%, *p* = 0.008, respectively).The mean TPR showed a significant decrease in SCH group than in control group (mean 975.85 ± 159.33 mmHg.min/L Vs mean 1120.24 ± 135.15 mmHg.min/L, *p* = 0.045). The results of FMD of the brachial artery revealed that there was a significant decrease in mean FMD in patients with SCH compared to euthyroid controls (mean 7.03 ± 4.02% Vs mean 13.48 ± 4.57%, *p* = 0.003). (Table 2)

### Linear regression analysis for the factors affecting RAD in SCH patients

TSH, FT4, FT3, gland volume, echogenicity, CO, and TPR appeared to be significantly correlated with RAD by univariate analysis of variables. The multivariate regression analysis model showed that only higher FT4 and lower FMD were still significant independent factors controlling RAD in SCH patients (Table 3).

**Table 3:** Univariate and multivariate regression model identifies association of thyroid and cardiovascular variables with RAD in the SCH group

*Parameters*	*Univariate analysis*		*Multivariate analysis*	
	OR (95% CI)	*p*	OR (95% CI)	*p*
*FMD*	15.86 (13.85 ± 17.87)	0.001	12.68 (10.88 ± 14.49)	^a^0.032
*CO*	7.1 (6.3 ± 7.9)	0.013	8.45 (7.77 ± 9.14)	0.346
*TPR*	1100.99 (988.61 ± 1213.37)	0.028	965.85 (869.16 ± 1062.54)	0.073
*FREE T3*	5.49 (3.98 ± 7.01)	0.038	6.85 (5.55 ± 8.15)	0.176
*FREE T4*	2.03 (1.73 ± 2.32)	0.010	2.39 (2.13 ± 2.64)	80.04
*TSH*	0.84 (0.43 ± 1.26)	0.044	0.02 (−0.33 ± 0.38)	0.135
*thyroid U/S*				
*gland vol*	15.7 (8.09 ± 23.32)	0.005	30.52 (23.96 ± 37.07)	0.073
*Vascularity*	0.86 (0.41 ± 1.31)	0.012	1.64 (1.25 ± 2.02)	0.085
*nodularity*	0 (0.15 ± 0.18)	0.025	0.27 (−0.12 ± 0.42)	0.205
*echogenicity*	−0.57 (−0.86 ± −0.28)	0.006	0.07 (−0.27 ± 0.41)	0.853

*P* *p* value for comparing between the studied groups

*CO* Cardiac output, *TPR* Total peripheral resistance, *FMD* Flow mediated dilatation, *RAD* Renal artery distensibility

^a^Statistically significant at *p* ≤ 0.05

### Multivariate analysis for the factors affecting glomerular (eGFR) and tubular (FeNa) functions in SCH patients

**Fig. 1:**
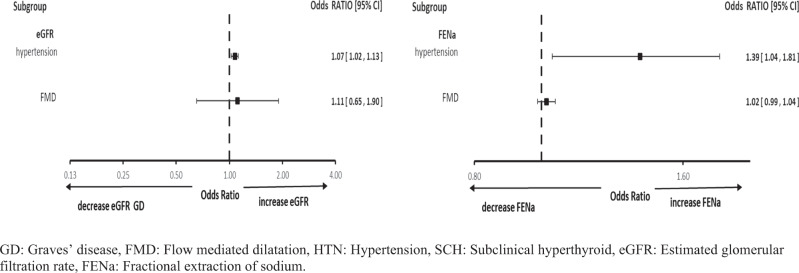
Multivariate regression analysis for FMD and FENa in the SCH group. GD: Graves’ disease, FMD: Flow mediated dilatation, HTN: Hypertension, SCH: Subclinical hyperthyroid, eGFR: Estimated glomerular filtration rate, FENa: Fractional extraction of sodium

Multivariate analysis was performed in the SCH group for variables affecting eGFR and FeNa (variables with a *p* value < 0.20 in the univariate analysis). After adjustment for age, TSH, FT3, FT4, CO and presence of HTN. It was found that HTN is the only significant factor that affected eGFR and FeNa in SCH patients (*p* = 0.0001 and 0.011, respectively) without effect on FMD (Fig. 1).

## Discussion

Thyroid dysfunction is known to induce noticeable changes in both glomerular and tubular functions of the kidney as well as affects electrolyte and water balance [25].

Meanwhile, several studies have investigated the relationship between subclinical hypothyroidism and renal function, only very few studies have addressed such a relationship with SCH. In fact, the studies that addressed SCH and kidney function in relation to hypertension and endothelial dysfunction are scarce. In this study, we investigated the effect of SCH on kidney function and vasculature. The major finding of our study is that the renal function changes in patients with SCH could be affected both directly by thyroid hormones causing vascular resistance reduction & lowering RAD and indirectly through hypertension.

The results of the present study showed that serum creatinine was significantly lower while eGFR and BUN were significantly higher in the SCH group compared to the control group. Our results agreed with those of Lippi et al. [26] who studied thyroid status and renal function in a general population of 13,383 unselected outpatients. They found that patients with TSH < 0.2 mIU/L (SCH) had increased eGFR, while those with TSH > 2.5 mIU/L had decreased eGFR. Moreover, they concluded that TSH levels were an independent predictor of eGFR and speculated that it might also be advisable to periodically evaluate the renal function by a simple parameter such as the eGFR in patients with thyroid dysfunction. This also was in line with Wang X et al. [27] who showed, in their meta-analysis, that hyperthyroidism in its early stages (SCH) is associated with decreased serum creatinine and increased GFR. In addition, they found a non-significant association between SCH with CKD in both cross-sectional and cohort study subgroups which further supports our findings of the low serum creatinine in patients with SCH. Also, Englund Flodström et al [28]. showed a significant inverse relation between FT3 and FT4 and plasma creatinine while they found a significant positive relation between TSH and plasma creatinine concentration among hyperthyroid patients. They described their results to further support the proportional inverse relation between thyroid hormone activity and plasma creatinine concentration. In agreement with our results, Kim et al. [29] demonstrated an inverse relationship between TSH levels and eGFR. Our findings of low creatinine and high eGFR among SCH patients can be explained by the glomerular hyperfiltration present in those patients due to the early hyperthyroid state resulting in increased eGFR and decreased serum creatinine, while increased BUN can be due to the presence of a relative dehydration state resulting from polyuria in those patients.

Although, in overt hyperthyroidism, the enhanced tubular reabsorption of sodium due to increased activity of Na+/H+ exchanger in combination with the decreased load of filtered sodium causes a decrease in the pressure-diuresis-natriuresis response [30], we did not find a significant difference in electrolytes whether in blood or urine among SCH patients. This is consistent with a previous study that compared patients with SCH and euthyroidism regarding the mean plasma levels of electrolytes and did not reveal any significant differences [31].

We reported an no significant differences in ARR in both SCH and euthyroid groups. In conjunction with our result, Marcisz et al. [32] showed that the basal PRA in patients with hyperthyroidism was found to be similar to that of the healthy controls. There was no relationship between the severity of hyperthyroidism and PRA.

Hyperthyroidism, even in the subclinical form, is known to produce manifest cardiovascular changes and alter cardiovascular hemodynamics [33, 34]. The results of our study showed that the pulse, CO, EF, and FS were significantly increased in SCH compared to the control group. On the contrary, Rodondi et al. [35] demonstrated a statistically significant decrease in EF between SCH patients and euthyroid controls, but without increased risk of heart failure after 12 years of follow up, yet their study population were elderly (age ≥ 65 years). Our results agree with those of different studies shown in the review article by Biondi et al. [36] who reported increased pulse and FS in patients with SCH than in euthyroid subjects which could be explained by the direct effect of thyroid hormones.

Our current results showed a high prevalence of HTN among SCH patients. Our results agreed with that of Walsh et al. [37] who reported increased frequency of HTN among patients with SCH and Kaminski et al. [14] who performed 24 h ambulatory BP monitoring for patients with SCH and found a higher nocturnal mean systolic and diastolic BP and higher mean BP compared to euthyroid controls. High BP in patients with SCH can be explained by the elevated CO and the increased blood volume [38].

Both overt and subclinical hypothyroidism are well-known to be established risk factors for endothelial dysfunction [39]. Yet, the relationship between subclinical hyperthyroidism and endothelial dysfunction is still to be determined. Our current results showed that FMD was significantly lower in the SCH group than in the control group and 85.71% of patients in the SCH showed abnormal FMD indicating the presence of endothelial dysfunction in patients with SCH. Likewise, Cikim et al. [40] showed that FMD was significantly lower in patients with SCH than in euthyroid subjects. Moreover, our results showed that both RAD and TPR were significantly lower in the SCH group compared to the control group. In line with our results, Vargas-Uricoechea et al. [41] showed that hyperthyroidism is associated with a reduced peripheral vascular resistance. This can be explained by the action of thyroid hormones on the vascular endothelium via stimulating the production of endothelial nitric oxide, leading to a reduction in systemic vascular resistance. For several years, T3 is known as a potent direct vasodilator, T3 induces vascular relaxation which is attributed to endothelium-dependent mechanisms or the vascular smooth muscle cell (VSMC)-related mechanisms mediating such vasodilatory effect [42].

Hence, SCH is associated with reduced TPR, FMD & RAD which reflected a reduced vascular tone and a stable stiffness. This explains our results that the high BP and thyroid hormones could be the main driving force for renal function changes among patients with SCH without the direct effect of renal vasodysfunction nor the effect of endothelial dysfunction. In accordance with our results and to prove our findings, one study showed that when studying the effect of endothelial dysfunction on renal vascular response in isolated perfused kidneys from rats with thyroid dysfunction, the renal vascular response to angiotensin II was found to be normal and is not influenced by the endothelial relaxing factors or by endothelium removal [43].

To the best of our knowledge, we are the first to investigate the effect of SCH on kidney function and vasculature in one study and postulate a novel mechanism for kidney function changes in patients with SCH. The main strength of our study is the large sample size & less selection bias (being from the whole population). However, the present study has two limitations. First, given the case-control and observational design, it is not possible to infer causality from the associations described. Secondly, the effects of thyroid autoantibodies (TRAb) and the duration of SCH were not taken into consideration. Our study can be the basis for further prospective studies in this context.

## Conclusion

In patients with SCH, the main driving mechanisms behind changes in renal glomerular and tubular functions could be directly through vascular resistance reduction as a direct effect of thyroid hormones and indirectly through hypertension. Our study may add a novel postulated mechanism for kidney function changes in patients with SCH. Hence, the impact of SCH on the kidney should be reconsidered which may influence the interpretation of renal function status and may affect treatment decisions in the setting of SCH.

## Data Availability

Statement institutional approval was given for the analysis and reporting of anonymized data collected as part of routine clinical care, but we do not have consent from patients to make the data set publicly available.
